# Deep Learning Applied to 12-Lead ECGs for Detection of Structural, Metabolic and Systemic Disease

**DOI:** 10.3390/diagnostics16172834

**Published:** 2026-09-03

**Authors:** Shlomo Shaulian, Roman Zeltser, Amgad N. Makaryus

**Affiliations:** 1 Long Island Campus, New York Institute of Technology College of Osteopathic Medicine, Old Westbury, NY 11568, USA; sshaulia@nyit.edu; 2 Department of Cardiology, Northwell and Northwell Cardiovascular Institute, Donald and Barbara Zucker School of Medicine at Hofstra/Northwell, New Hyde Park, NY 11042, USA; rzeltser@northwell.edu

**Keywords:** artificial intelligence, deep learning, electrocardiography, AI-ECG, structural heart disease, digital biomarkers

## Abstract

The 12-lead electrocardiogram (ECG) is inexpensive, noninvasive, and widely available, but conventional interpretation may not capture subtle signals related to cardiac structure, systemic physiology, and future risk. This review examines the use of deep learning–enabled ECG analysis beyond conventional arrhythmia detection and evaluates the maturity, clinical relevance, and implementation challenges of these applications. This narrative review synthesizes landmark studies, external validation cohorts, pragmatic implementation trials, and recent investigations of artificial intelligence–enabled ECG (AI-ECG) models for structural, metabolic, systemic, and prognostic assessment. Particular attention is given to model performance, validation, clinical actionability, and barriers to translation. AI-ECG models have demonstrated the ability to detect reduced left ventricular ejection fraction, hypertrophic cardiomyopathy, valvular disease, cardiac amyloidosis, pulmonary hypertension, hyperkalemia and other dyskalemias, hyperthyroidism, anemia, and sepsis, and to estimate biologic age and mortality risk. Evidence is most mature for screening for reduced left ventricular ejection fraction, supported by large derivation cohorts, external validation, prognostic follow-up, and the EAGLE pragmatic trial. Evidence for many other applications remains retrospective or exploratory, with limitations related to reference standards, generalizability, calibration, disease prevalence, interpretability, and the absence of clearly defined clinical pathways. AI-ECG has the potential to expand the ECG from a conventional diagnostic test into a screening and decision-support platform. Its near-term role is to identify patients who may benefit from confirmatory imaging, laboratory testing, rhythm monitoring, or specialist evaluation. Broader adoption will require prospective validation, workflow integration, subgroup assessment, post-deployment monitoring, regulatory oversight, and evidence that AI-guided care improves outcomes.

## 1. Introduction

The 12-lead electrocardiogram (ECG) is inexpensive, noninvasive, widely available, and has been used for more than a century to record cardiac electrical activity. It is traditionally interpreted to diagnose arrhythmias, detect myocardial ischemia and infarction, evaluate pacemaker and implantable-device function, and assess electrolyte abnormalities [1]. Conventional interpretation relies on predefined waveform characteristics and rule-based pattern recognition, which may miss subtler physiologic and pathologic signals [2].

Recent advances in artificial intelligence (AI), particularly deep learning, have transformed the analysis of complex medical data. Unlike traditional rule-based approaches, deep learning models can identify intricate patterns within large datasets without requiring explicit feature selection [3]. Emerging evidence suggests that ECG waveforms contain latent signatures of ventricular dysfunction [4], structural heart disease [5], metabolic abnormalities [6], and broader physiologic characteristics such as biological age [7] and future mortality risk [8].

These findings have expanded the view of the ECG from a tool primarily used for rhythm assessment to a potential platform for disease screening, risk stratification, and earlier diagnosis. This review examines artificial intelligence-enabled ECG (AI-ECG) applications beyond arrhythmia detection, highlights the studies that first established these hidden disease signatures within ECG waveforms, evaluates current and emerging clinical applications, and discusses the challenges associated with clinical implementation. Unlike reviews centered on arrhythmia classification or on a single condition, it emphasizes structural, metabolic, and systemic applications and the practical requirements for translating them into clinical care.

### Literature Search Strategy

A targeted narrative literature search was conducted using PubMed/MEDLINE and Google Scholar to identify studies examining artificial intelligence and deep learning applications to 12-lead electrocardiography beyond conventional arrhythmia detection. English-language articles published from January 2015 through August 2026 were considered. Search terms included combinations of “artificial intelligence,” “deep learning,” “machine learning,” “electrocardiogram,” “ECG,” “12-lead ECG,” and “AI-ECG,” together with terms related to the major applications discussed in this review, including ventricular dysfunction, heart failure, structural heart disease, hypertrophic cardiomyopathy, valvular disease, cardiac amyloidosis, pulmonary hypertension, hyperkalemia, biologic age, mortality prediction, and clinical implementation. Reference lists of relevant primary studies and review articles were also examined to identify additional publications. Priority was given to peer-reviewed original studies, external validation studies, prospective or pragmatic implementation studies, and relevant consensus or reporting guidelines. Studies focused exclusively on conventional arrhythmia classification were generally excluded unless they provided important context for AI-ECG development. Because this was a narrative rather than systematic review, formal risk-of-bias assessment and quantitative meta-analysis were not performed. The final search was conducted on 21 August 2026.

## 2. How Deep Learning Extracts Additional Information from ECGs

### 2.1. Traditional ECG Interpretation

Traditional ECG interpretation is based on the analysis of predefined waveform characteristics and recognizable electrocardiographic patterns. Clinicians evaluate features such as heart rate and rhythm, P-wave morphology, PR interval, QRS duration and morphology, cardiac axis, ST-segment deviation, T-wave abnormalities, and QT interval to identify arrhythmias, conduction disturbances, myocardial ischemia, chamber enlargement, and other cardiovascular abnormalities [2].

Conventional computerized ECG analysis similarly extracts selected waveform features and applies predefined diagnostic criteria. Although this approach has improved standardization, it remains limited by the features and rules programmed into the system. Despite these limitations, the standard 12-lead ECG remains a fundamental and widely used cardiovascular diagnostic tool, and conventional computerized analysis continues to provide rapid and clinically useful measurements and preliminary interpretations in routine practice. Classical signal-processing and rule-based algorithms therefore remain an important component of contemporary electrocardiography. Deep-learning approaches should be viewed as complementary tools that may extract additional information from ECG waveforms rather than as replacements for established ECG acquisition, processing, interpretation, or physician review [2]. Consequently, both human and computerized interpretation may overlook subtle or distributed signals related to cardiac structure, function, and systemic physiology [5].

### 2.2. Deep Learning

Unlike traditional ECG interpretation, which relies on predefined waveform measurements and diagnostic rules, deep learning models learn directly from raw ECG signals. Using artificial neural networks trained on large datasets containing hundreds of thousands to millions of ECGs, these systems can automatically identify complex relationships within waveform data without requiring manual feature selection.

This approach has enabled a transition from rule-based analysis to end-to-end pattern recognition. Rather than focusing solely on established ECG features such as intervals, amplitudes, and segment morphology, deep learning models analyze the entire waveform and can detect subtle patterns distributed across multiple leads [9]. As a result, AI systems may identify clinically relevant information that is not readily apparent through conventional interpretation or visual inspection by clinicians [4]. A handful of influential studies, described next, showed how much diagnostic and prognostic information this makes accessible.

### 2.3. Deep Learning Architectures Used in AI-ECG

Although convolutional neural networks (CNNs) predominate in AI-ECG research, the architectures used across studies vary substantially. Standard CNNs learn local temporal and morphologic features directly from ECG waveforms, while deeper residual networks incorporate skip connections that facilitate the training of more complex models. Other architectures, such as ECG12Net, combine dense connectivity with attention mechanisms to integrate information across individual ECG leads. The major characteristics, advantages, and limitations of representative architectures used in AI-ECG studies are summarized in Table 1. These architectural differences can affect feature extraction, computational requirements, and interpretability. However, reported performance cannot be directly compared across architectures because studies differ substantially in target condition, dataset composition, sample size, preprocessing, and validation strategy. Therefore, current evidence does not establish a universally superior deep learning architecture for AI-ECG analysis.

## 3. Landmark Studies Establishing the Field

### 3.1. Detection of Left Ventricular Dysfunction

The field effectively began with left ventricular systolic dysfunction. In 2019, Attia et al. trained a convolutional neural network on paired ECG and echocardiographic data to identify patients with a left ventricular ejection fraction (LVEF) ≤ 35%, using over 44,000 patients for model development and more than 52,000 for testing. The model achieved an area under the receiver operating characteristic curve (AUC) of 0.93, with a sensitivity of 86.3% and specificity of 85.7%, a strong performance for a test this cheap and widely available [4].

The more striking implication was that a routine ECG carries information about cardiac structure that a human reader cannot extract from the same tracing. Notably, patients with a positive AI-ECG despite a preserved ejection fraction were more likely to develop ventricular dysfunction during follow-up, suggesting that the model may be identifying subclinical disease rather than only established systolic dysfunction, positioning AI-ECG as a screening tool for both current and future ventricular dysfunction.

### 3.2. Prediction of Future Atrial Fibrillation

AI-ECG was subsequently applied to sinus-rhythm ECGs to identify patients at risk for atrial fibrillation (AF). Traditionally, AF is diagnosed when the arrhythmia is directly captured on an ECG or rhythm monitor. However, Attia et al. demonstrated that an AI-enabled algorithm could identify AF-associated patterns from standard 12-lead ECGs recorded during sinus rhythm, suggesting that the ECG may contain latent signatures of atrial remodeling or electrophysiologic vulnerability even when AF is not present on the tracing itself [12].

This marked a conceptual shift: AI-ECG moved from recognizing an arrhythmia already visible on the tracing to flagging a patient’s underlying risk of developing one. Subsequent studies extended this concept by using deep learning models to predict new-onset AF from routine 12-lead ECGs. Raghunath et al. showed that AI-ECG prediction of future AF could also enrich for patients at risk of AF-related stroke, supporting the potential use of these models for targeted screening and rhythm monitoring [13].

Because AF may remain intermittent and undetected until complications such as stroke occur, AI-ECG could help select patients for ambulatory monitoring or closer rhythm surveillance. However, prospective studies are still needed to determine whether AI-guided monitoring improves AF detection or clinical outcomes before routine implementation [14].

### 3.3. Detection of Hyperkalemia and Other Systemic Conditions

Early studies of AI-based hyperkalemia detection demonstrated that ECG waveforms may contain clinically useful biochemical information. Hyperkalemia is known to affect cardiac conduction and repolarization, but conventional ECG changes can be inconsistent and may be absent even when serum potassium is dangerously elevated. In a 2019 study, Galloway et al. developed a deep-learning model to screen for hyperkalemia using ECG data from patients with renal disease. The model was trained on more than 1.5 million ECGs from over 449,000 patients and, using only leads I and II, detected hyperkalemia with AUC values ranging from 0.853 to 0.883 across validation sites [6].

This study expanded AI-ECG by suggesting that ECG waveforms could support noninvasive screening for a metabolic abnormality ordinarily diagnosed through laboratory testing. Later work carried the approach to 12-lead detection of both hypokalemia and hyperkalemia [11] and point-of-care potassium estimation [15].

In emergency, dialysis, renal, or remote-care settings, an AI-ECG alert could prioritize confirmatory testing when laboratory results are delayed or repeated blood sampling is impractical. These models should not replace serum potassium measurement or clinical judgment.

Reports of AI-ECG detection of severe dyscalcemia [16] further suggest that deep learning may recognize cardiovascular effects of systemic disease.

## 4. Current Clinical Applications Beyond Arrhythmias

### 4.1. Ventricular Dysfunction and Heart Failure

Building on the landmark work described above, subsequent studies have evaluated whether AI-ECG detection of ventricular dysfunction generalizes across clinical populations and can be incorporated into real-world screening pathways. Kashou et al. studied a community-based cohort and reported strong performance for detecting preclinical left ventricular systolic dysfunction, supporting the potential role of AI-ECG in upstream screening before overt heart failure is recognized [17]. Adedinsewo et al. evaluated an AI-enabled ECG algorithm in emergency department patients presenting with dyspnea, showing that AI-ECG may also assist in triage settings where rapid identification of cardiac dysfunction can guide additional testing [18]. Read together, these studies point to a dual role: opportunistic screening in asymptomatic patients, and evaluation of symptomatic patients in whom ventricular dysfunction is part of the differential.

A major step toward clinical implementation came with the EAGLE trial, a pragmatic cluster-randomized trial evaluating AI-guided screening for low ejection fraction in primary care. In this study, AI-ECG results were made available to clinicians for patients without previously known heart failure. The intervention increased new diagnoses of low ejection fraction within 90 days, particularly among patients with a positive AI screen, and increased targeted echocardiography in the AI-positive subgroup without substantially increasing echocardiography use across the entire population [19]. Unlike most retrospective AI-ECG studies, the EAGLE trial demonstrated that model output could alter real-world diagnostic behavior. However, the trial primarily established increased case identification and targeted echocardiography; whether this approach improves treatment initiation, long-term outcomes, or cost-effectiveness remains uncertain.

Real-world and external validation studies have further clarified both the strengths and limitations of AI-ECG screening for ventricular dysfunction. Harmon et al. reported stable performance of an AI-ECG model across time and demographic subgroups, addressing concerns about temporal drift and subgroup bias [20]. König et al. externally validated an existing AI-ECG algorithm in a European cohort and found good overall discrimination for left ventricular systolic dysfunction, although performance was lower in certain subgroups, including patients with tachycardia, atrial fibrillation, or wide QRS complexes [21]. These findings emphasize that AI-ECG output should be interpreted in the context of rhythm, conduction abnormalities, and clinical setting rather than treated as a standalone diagnosis.

This echoes the earlier observation in systolic dysfunction: a positive AI-ECG in a patient with preserved ejection fraction may not always represent a simple false positive. In outcome-focused analyses, patients with abnormal AI-ECG results despite normal contemporaneous systolic function were more likely to have other structural abnormalities or worse outcomes compared with true-negative patients [22]. This suggests that AI-ECG may identify a broader latent cardiac phenotype rather than merely detect current low ejection fraction.

AI-ECG screening may also be valuable in settings where access to echocardiography is limited. A recent study in Kenyan outpatient facilities evaluated an AI-ECG algorithm for detecting left ventricular systolic dysfunction and reported high sensitivity and negative predictive value compared with echocardiography [23]. This type of performance is clinically relevant because a highly sensitive AI-ECG could help prioritize limited imaging resources for patients most likely to benefit from confirmatory echocardiography.

Compared with reduced ejection fraction, AI-ECG detection of heart failure with preserved ejection fraction (HFpEF) is less mature but increasingly important. HFpEF is diagnostically challenging because ejection fraction is preserved and diagnosis often requires integration of symptoms, echocardiographic measures of diastolic function, natriuretic peptides, and clinical risk factors. Kwon et al. developed a deep learning model for early detection of HFpEF using ECG data and reported promising discrimination in internal and external validation AUCs of 0.866 and 0.869, respectively [24]. More recent studies have extended this concept by using AI-ECG to predict left ventricular diastolic dysfunction and increased filling pressures [25] and HFpEF-related risk [26].

Of the applications reviewed here, reduced-ejection-fraction detection is the most clinically mature and the closest to routine use: the evidence spans large derivation cohorts, external validation, prognostic follow-up, and a pragmatic implementation trial. Nevertheless, performance varies across populations and may be affected by rhythm, conduction abnormalities, disease prevalence, and the threshold selected for a positive result. Evidence for HFpEF and diastolic dysfunction remains less mature, with greater heterogeneity in disease definitions and limited prospective evidence that AI-guided detection improves clinical outcomes.

### 4.2. Structural Heart Disease

Beyond ventricular dysfunction, AI-ECG models have been developed to identify structural disorders that ordinarily require echocardiography, cardiac magnetic resonance imaging, or disease-specific testing for confirmation. Hypertrophic cardiomyopathy (HCM) is one of the best-studied examples of AI-ECG detection of structural heart disease. In a landmark study, Ko et al. developed a convolutional neural network using standard 12-lead ECGs to detect HCM and reported strong diagnostic performance, with an AUC of 0.96, sensitivity of 87%, and specificity of 90% [10]. This was clinically important because HCM may be underdiagnosed, particularly in patients with subtle or nonspecific ECG findings, and earlier detection can prompt confirmatory imaging, family screening, risk stratification, and disease-specific management. Subsequent international external validation further supported the transportability of AI-ECG for HCM detection, with Siontis et al. reporting an AUC of 0.922 across cohorts from Bern, Oxford, and Seoul [27]. More recent real-world implementation studies suggest that AI-ECG-based HCM alerts can be deployed in routine care, although positive results still require clinical correlation and confirmatory imaging [28].

AI-ECG has also been applied to valvular heart disease, particularly aortic stenosis and mitral regurgitation. Kwon et al. developed a deep learning algorithm to detect aortic stenosis from ECGs, demonstrating that valvular disease may produce electrical signatures detectable beyond standard ECG criteria [29]. Cohen-Shelly et al. later evaluated AI-ECG screening for moderate-to-severe aortic stenosis in a large Mayo Clinic cohort and reported a sensitivity of 78% and specificity of 74%. As with the ejection-fraction models, patients with apparent false-positive AI-ECG results here may still represent a higher-risk group, as some later developed clinically significant aortic stenosis [30]. Larger multicenter work has expanded this approach beyond aortic stenosis alone. Vaid et al. applied deep learning to more than 600,000 ECG-echocardiogram pairs across five hospitals and externally validated models for both mitral regurgitation and aortic stenosis, supporting the broader role of AI-ECG for left-sided valvular dysfunction [31].

Cardiac amyloidosis is another clinically relevant application because diagnosis is often delayed, while earlier recognition has become increasingly important with the availability of disease-modifying therapy for transthyretin amyloid cardiomyopathy. Grogan et al. developed an AI-ECG model for early detection of cardiac amyloidosis, supporting the concept that infiltrative myocardial disease produces subtle ECG features that can be recognized computationally [32]. Goto et al. subsequently demonstrated a fully automated approach using ECGs and echocardiograms to detect cardiac amyloidosis, with strong internal and external performance [33]. A subsequent post-development validation study found that the AI-ECG model retained good overall discrimination, with an AUC of 0.84, although performance was lower in some subgroups, including Hispanic patients and patients with left ventricular hypertrophy or left bundle branch block [34]. These studies are important because amyloidosis can present with nonspecific symptoms and may not be suspected until advanced disease is present. AI-ECG may therefore help enrich the population selected for further evaluation with echocardiography, cardiac magnetic resonance imaging, nuclear scintigraphy, or serum and urine testing when clinical suspicion is present.

The strength of evidence differs across these structural applications. HCM has shown consistently high discrimination, international external validation, and early real-world implementation, making it one of the more reproducible structural AI-ECG targets. Evidence for valvular disease and cardiac amyloidosis is also encouraging but remains more dependent on retrospective cohorts assembled from patients who underwent both ECG and imaging. Such cohorts may introduce verification and referral bias and may not reflect performance in unselected screening populations. Moreover, because these disorders are relatively uncommon in general populations, high discrimination does not necessarily translate into a high positive predictive value. AI-ECG is therefore best viewed as a tool for selecting patients for confirmatory imaging rather than establishing a structural diagnosis independently.

### 4.3. Pulmonary Hypertension

Pulmonary hypertension (PH) has been studied as a potential target for AI-ECG detection, with models designed to identify electrical signatures of elevated pulmonary pressures and right-sided cardiac remodeling. Kwon et al. developed one of the earlier AI-ECG models for prediction of pulmonary hypertension using standard 12-lead ECGs and clinical variables. The model was trained using more than 56,000 ECGs and demonstrated strong diagnostic performance for PH in both internal and external validation cohorts, with reported AUC values of 0.859 and 0.902, respectively. Importantly, among patients without known PH at baseline, those classified as high risk by the AI model had a substantially higher subsequent incidence of PH than those classified as low risk [35]. This suggested that AI-ECG may not only identify existing pulmonary hypertension but may also detect early ECG signatures associated with future disease development.

Liu et al. further extended this concept by developing a deep learning ECG model to detect elevated pulmonary artery pressure estimated by echocardiography. Using paired ECG and echocardiographic data from more than 41,000 patients, the model identified elevated estimated pulmonary artery pressure with an AUC of approximately 0.88, along with sensitivity and specificity of about 81% and 80%, respectively. The model also performed consistently across important clinical subgroups and was externally validated in an independent cohort [36]. These findings support the possibility that routine ECGs may contain information reflecting pulmonary vascular load, right ventricular pressure overload, or secondary electrical changes caused by altered cardiopulmonary hemodynamics.

More recently, DuBrock et al. developed the Pulmonary Hypertension Early Detection Algorithm (PH-EDA), a deep learning model designed to identify patients likely to have PH using 12-lead ECG data. The model was trained and tested in a large Mayo Clinic cohort and externally validated in a Vanderbilt cohort. PH-EDA achieved strong discrimination at the time of PH diagnosis and also demonstrated predictive ability when applied to ECGs obtained months to years before clinical diagnosis. This finding is clinically important because delayed recognition of PH is common, and earlier identification may allow more timely echocardiography, specialist referral, and confirmatory hemodynamic testing when appropriate [37].

However, several limitations remain. Many AI-ECG studies in pulmonary hypertension use echocardiographic estimates of pulmonary artery pressure as the reference standard, even though right heart catheterization remains the diagnostic gold standard. In addition, ECG patterns associated with PH may overlap with those caused by left-sided heart disease, chronic lung disease, conduction abnormalities, atrial fibrillation, or other causes of right ventricular strain. Since PH is also relatively uncommon in general ECG populations, positive predictive value may be limited when AI-ECG is applied broadly. Overall, the pulmonary hypertension literature shows consistent discrimination across multiple cohorts and suggests that AI-ECG may identify disease before formal diagnosis. However, the evidence remains less clinically mature than that for reduced ejection fraction because most studies are retrospective, reference standards are inconsistent, and no prospective trial has demonstrated that AI-guided screening reduces diagnostic delay or improves patient outcomes without producing excessive downstream testing.

### 4.4. Metabolic and Systemic Conditions

The early hyperkalemia work opened the door to broader dyskalemia estimation and to other systemic physiologic states. Lin et al. developed a point-of-care AI-enabled ECG model to estimate serum potassium and detect both hypokalemia and hyperkalemia in emergency department patients. In a cohort including more than 34,000 emergency department visits with paired ECG and laboratory potassium measurements, the model achieved AUC values greater than 0.95 for moderate-to-severe hyperkalemia and greater than 0.85 for moderate-to-severe hypokalemia. Patients with discordance between laboratory and AI-predicted ECG potassium also had worse outcomes, suggesting that AI-ECG potassium signals may reflect not only serum electrolyte concentration but also underlying physiologic vulnerability [15].

Biologic age estimation represents another important systemic application of AI-ECG. Rather than detecting a single disease state, these models attempt to estimate a patient’s physiologic or “ECG age” from raw ECG waveforms. Lima et al. trained a deep neural network to estimate age from 12-lead ECGs in the CODE study cohort, which included more than 1.5 million patients. Patients whose AI-estimated ECG age was more than eight years older than their chronological age had significantly higher mortality, while those whose ECG age was more than eight years younger had lower mortality. This association persisted even among individuals with apparently normal ECGs, suggesting that AI-derived ECG age may capture latent cardiovascular health or physiologic reserve not apparent through standard interpretation [7].

AI-ECG has also been used for direct mortality prediction. Raghunath et al. trained a deep neural network using more than 1.1 million 12-lead ECGs from over 250,000 patients to predict one-year all-cause mortality. The model achieved an AUC of 0.88 in the held-out test set and maintained strong performance even among ECGs interpreted as normal by physicians, with an AUC of 0.85. Cardiologists reviewing selected normal ECGs were unable to reliably identify the mortality-associated patterns detected by the model [8]. This study demonstrated that AI-ECG can extract prognostic information from ECG waveforms that may not be visually apparent, reinforcing the concept of the ECG as a digital biomarker for future risk.

Other emerging systemic applications include endocrine, hematologic, and inflammatory conditions. Deep learning models have been developed to detect overt hyperthyroidism from ECGs, with one multicenter study reporting AUCs of 0.926 for internal validation and 0.883 for external validation using 12-lead ECGs [38]. A later study also found that AI-ECG detection of hyperthyroidism was associated with increased risk of mortality and new-onset heart failure [39]. AI-ECG has also been studied for noninvasive hemoglobin estimation, with a deep learning model detecting moderate-to-severe anemia with AUCs of 0.854 internally and 0.824 externally [40], and for sepsis screening, with reported AUCs of 0.901 internally and 0.863 externally using 12-lead ECGs [41].

Across these systemic applications, AI-ECG can clearly extract diagnostic and prognostic information beyond the heart itself. However, how such predictions should enter clinical screening and decision-making is far from settled. The evidence is strongest for dyskalemia because potassium-related models have been evaluated in large cohorts, across different clinical settings, and with laboratory measurements serving as an objective reference standard. Even so, variations in the interval between ECG acquisition and blood sampling, treatment administered before testing, renal function, and medication use may affect the relationship between the ECG signal and measured electrolyte concentration. Evidence for hyperthyroidism, anemia, and sepsis remains more exploratory, with fewer independent validations and no prospective evidence that AI-ECG screening improves management or outcomes. ECG-age and mortality models demonstrate strong prognostic associations but present an additional challenge: a risk estimate has limited clinical value unless it leads to a defined and beneficial action.

**Figure 1:**
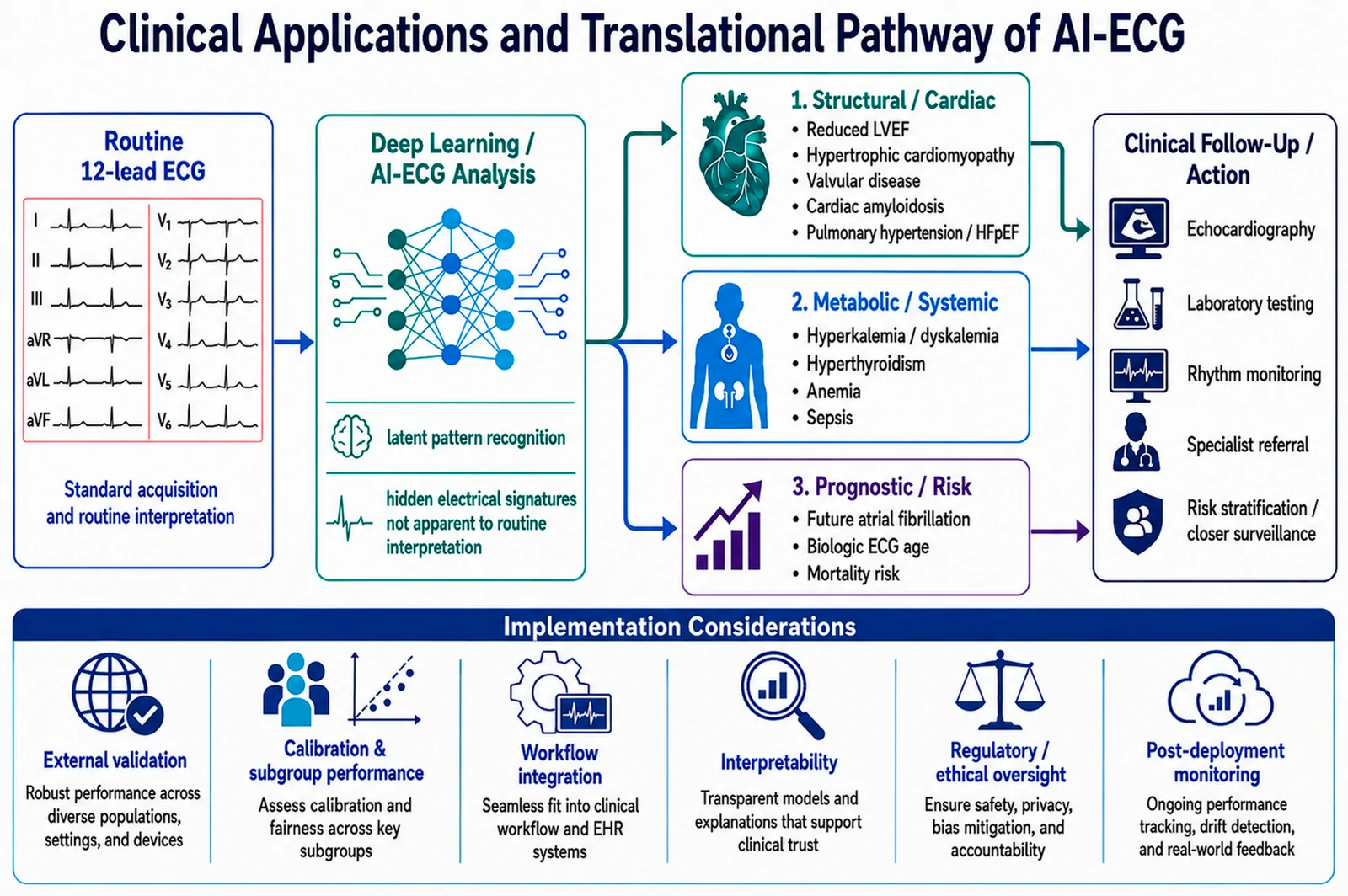
Clinical applications and translational pathway of artificial intelligence-enabled electrocardiography. Routine 12-lead ECGs can be analyzed using deep-learning models to identify latent signatures associated with structural and cardiac disease, metabolic and systemic conditions, and prognostic risk. AI-ECG outputs should function as clinical decision-support signals linked to appropriate confirmatory testing, monitoring, specialist referral, or risk stratification. Clinical implementation requires external validation, calibration and subgroup assessment, workflow integration, interpretability, regulatory and ethical oversight, and post-deployment monitoring.

The major applications of AI-ECG, their associated clinical follow-up pathways, and the key considerations required for implementation are summarized in Figure 1. Representative diagnostic performance metrics for major AI-ECG applications are summarized in Table 2.

The major clinical applications of AI-ECG and their potential roles are summarized in Table 3.

## 5. Clinical Implications

### 5.1. AI-ECG as a Screening Tool

AI-ECG may enable opportunistic screening by applying additional analysis to ECGs already obtained during outpatient visits, emergency evaluations, preoperative testing, and chronic disease follow-up. Evidence from reduced-ejection-fraction models and the EAGLE trial suggests that this approach can identify subclinical disease and influence diagnostic evaluation [4,19]. EchoNext broadened the same idea to structural abnormalities, again with echocardiography as the confirmatory pathway [42], while potassium models may prioritize urgent laboratory testing without replacing serum measurement [6,15].

Despite these advantages, AI-ECG screening requires careful implementation. A positive AI-ECG result is not a diagnosis; it is a risk signal that should prompt clinical correlation and, when appropriate, confirmatory testing. The usefulness of screening depends heavily on disease prevalence, because even models with strong discrimination may have modest positive predictive value in low-prevalence populations. False positives may lead to additional testing, patient anxiety, and increased resource use, while false negatives could create inappropriate reassurance if clinicians rely on AI output over clinical judgment.

Accordingly, the value of AI-ECG screening should be judged by net clinical benefit rather than diagnostic yield alone. This benefit depends on disease prevalence, the availability of an effective confirmatory test and treatment, the capacity of the health system to evaluate positive results, and the consequences of false-positive and false-negative classifications. The evidence is therefore most persuasive when AI-ECG targets a condition such as reduced ejection fraction, for which a positive screen can lead to echocardiography and established treatment. Its clinical value is less certain for broad prognostic outputs when no standardized intervention follows a high-risk result. Prospective studies should report time to diagnosis, treatment initiation, patient outcomes, downstream testing, and cost-effectiveness rather than only the number of additional cases detected.

### 5.2. Accessibility and Scalability

AI-ECG has the potential to scale because it adds computational analysis to a relatively inexpensive test already embedded in routine care. Unlike a new imaging or laboratory program, it can be applied to ECGs obtained across outpatient, emergency, inpatient, and preoperative settings. However, scalability is not automatic and depends on digital waveform storage, compatible ECG systems, software integration, technical support, and an available pathway for responding to positive results.

Validation in Kenyan outpatient facilities illustrates how AI-ECG could help prioritize scarce echocardiography and specialist resources, and it may extend cardiovascular screening beyond tertiary centers in settings where treatable disease is frequently recognized late [23].

Economic analyses have begun to evaluate AI-ECG, although cost-effectiveness depends on disease prevalence, alert thresholds, implementation costs, and downstream testing. Its economic value will depend less on the marginal cost of running the algorithm than on whether it improves the targeting of confirmatory testing and patient outcomes [43].

Accessibility therefore depends on more than the availability of the algorithm. A health system may be able to generate an AI-ECG risk score yet lack timely echocardiography, laboratory testing, specialist referral, or treatment capacity to act on the result. Models developed in tertiary centers may also require local validation or recalibration before use in populations with different disease prevalence, ECG hardware, and referral patterns. Economic evaluations should account not only for the low marginal cost of running an algorithm, but also for software integration, maintenance, confirmatory testing, false-positive evaluations, workforce demands, and the possibility that implementation could widen disparities between well-resourced and under-resourced settings.

### 5.3. Integration into Clinical Practice

Successful integration of AI-ECG analysis into clinical practice requires more than strong model performance. AI-ECG systems must be incorporated into workflows in a way that is clinically useful, understandable to physicians, and linked to clear follow-up actions. A positive AI-ECG result should not be treated as a final diagnosis; rather, it should function as a clinical decision support signal that prompts physician review, clinical correlation, and confirmatory testing when appropriate. The practical value of AI-ECG therefore depends on how the result is delivered, who receives it, how urgent the alert is, and what diagnostic or referral pathway follows.

The EAGLE trial, discussed above, provides an early example of this model: AI results were delivered through the electronic health record (EHR) while clinicians retained responsibility for ordering confirmatory echocardiography [19].

Workflow design should vary according to the clinical setting and target condition. In broad outpatient screening, thresholds may need to favor specificity to avoid excessive false positives and unnecessary testing. In symptomatic or high-risk populations, such as patients with dyspnea, renal disease, suspected heart failure, or risk factors for pulmonary hypertension, a higher-sensitivity approach may be more appropriate because missing disease could have greater clinical consequences. Regardless of the threshold chosen, AI-ECG results should be paired with a defined clinical pathway, such as echocardiography for suspected ventricular dysfunction or structural heart disease, laboratory testing for suspected metabolic abnormalities, ambulatory monitoring for arrhythmia risk, or specialist referral for suspected pulmonary hypertension or infiltrative cardiomyopathy.

Physician oversight remains essential because symptoms, comorbidities, ECG quality, rhythm, and conduction abnormalities may affect the meaning of a model output. Reports should therefore present the predicted condition, probability or risk level, relevant limitations, and an appropriate next step without displacing clinical judgment [44].

Clinician acceptance represents another important barrier to implementation. Surveys of cardiologists suggest generally favorable views regarding the potential of AI to improve efficiency and patient care, but adoption remains uneven because of concerns regarding model validation, transparency, unfamiliarity with AI methods, and trust in automated recommendations [45]. These concerns may appropriately slow adoption, but the opposite problem—overreliance on AI output—also requires attention. Automation bias can occur when clinicians accept an automated recommendation without sufficient independent evaluation, particularly when the system is perceived as highly accurate or simplifies a complex decision [46]. AI-ECG systems should therefore be designed and implemented to preserve independent clinical assessment, require appropriate confirmatory testing, and make clear that model output is decision support rather than a definitive diagnosis.

Alert fatigue is a related and underappreciated risk. If AI-ECG alerts are too frequent, poorly targeted, or disconnected from actionable next steps, clinicians may ignore them or view them as burdensome. To avoid this, health systems should decide in advance whether AI results appear in the ECG report, EHR inbox, clinical note, order-entry system, or separate dashboard. They should also determine who is responsible for responding to the alert, how quickly follow-up should occur, and how actions should be documented. Embedding AI-ECG into existing order sets, referral pathways, or echocardiography workflows may help convert model predictions into practical clinical action.

Implementation should also include local validation and post-deployment monitoring for model drift, subgroup performance, downstream testing, and patient outcomes, further discussed in Section 6 [47].

Current evidence regarding AI-ECG workflow integration remains limited. The EAGLE trial showed that results delivered through the EHR can increase detection and targeted echocardiography, but it did not establish the optimal alert format, recipient, timing, threshold, or follow-up pathway. Future implementation studies should measure whether clinicians acknowledge and act on alerts, how quickly confirmatory testing is completed, whether treatment changes, and whether the system improves patient outcomes without creating excessive alert burden. Without clearly assigned responsibility and measurable follow-up, even an accurate model may produce an ignored or clinically unactionable result.

## 6. Challenges and Limitations

### 6.1. Interpretability

Interpretability is probably the limitation raised most often against deep-learning ECG analysis. Many high-performing AI-ECG models function as “black boxes,” meaning that the relationship between the input ECG waveform and the final prediction is not easily understood by clinicians. This creates practical challenges for clinical adoption, since physicians may be hesitant to act on a prediction if they cannot determine whether the model is relying on physiologically meaningful ECG features or on spurious correlations, artifacts, or dataset-specific patterns. Interpretability is particularly important when AI-ECG is used for conditions that are not traditionally diagnosed by ECG, such as reduced ejection fraction, pulmonary hypertension, hyperkalemia, or mortality risk.

Several explainability methods have been applied to ECG deep learning models, including saliency maps, gradient-weighted class activation mapping (Grad-CAM), integrated gradients, local interpretable model-agnostic explanations (LIME), Shapley additive explanations (SHAP), and concept-based approaches. These methods attempt to identify which portions of the ECG contributed most strongly to a model’s prediction. For example, saliency mapping in AI-ECG detection of hypertrophic cardiomyopathy has highlighted regions concentrated primarily in ventricular repolarization and ST–T segments [48]. Other work has used broader explainability frameworks to compare model attention with known cardiologist decision rules and to investigate whether deep learning models use clinically plausible ECG features [49].

However, explainability tools should be interpreted cautiously. Attribution maps can be unstable across models, sensitive to preprocessing choices, and may not faithfully represent the true causal basis of a model’s prediction. A highlighted waveform segment does not necessarily prove that the model is using a clinically valid feature, and different explainability methods may produce different results for the same ECG. Therefore, visual explanations should be considered hypothesis-generating rather than definitive evidence of model reasoning. Recent work evaluating attribution methods for ECG classification has emphasized the need for sanity checks, rigorous validation, and caution when using post-hoc explanations in high-stakes clinical settings [50].

Interpretability should not be equated with trustworthiness. A clinically plausible saliency map does not establish that a model is well calibrated, unbiased, generalizable, or using a causal disease signal. For deployment decisions, external validation, subgroup performance, calibration, uncertainty estimates, failure-mode testing, and post-deployment monitoring may be more important than a visually convincing explanation. Explainability methods remain useful for detecting artifacts, comparing model attention with established ECG knowledge, and investigating unexpected errors, but they cannot substitute for prospective clinical validation.

### 6.2. Dataset Bias, Generalizability, and Validation

AI-ECG models are highly dependent on the data used for training and validation. Dataset bias can occur when training populations differ from the patients in whom the model is later applied. This may involve differences in age, sex, race and ethnicity, disease prevalence, comorbidities, ECG acquisition systems, referral patterns, or the quality of reference labels such as echocardiographic measurements or laboratory values. If a model is trained primarily on data from a single health system, tertiary referral center, or highly selected patient population, it may perform less reliably in community clinics, international cohorts, or underrepresented groups [51].

Subgroup performance has drawn particular attention. Noseworthy et al. evaluated the effect of race on an AI-ECG model for left ventricular systolic dysfunction and emphasized the need to assess performance across demographic subgroups rather than relying only on overall accuracy [52]. More recent work has shown that deep learning models can distinguish ECGs from healthy Black and White participants, with analyses suggesting that the detected differences were influenced largely by non-genetic factors [53]. These findings do not mean that AI-ECG models are inherently biased, but they demonstrate why fairness testing and transparent subgroup reporting are necessary.

Generalizability is also affected by differences between derivation and external validation cohorts. For example, external validation of an AI-ECG algorithm for left ventricular systolic dysfunction in a population-based cohort demonstrated lower performance than the original internal validation, and the original decision threshold produced low sensitivity in the new population. This suggested that population-specific thresholds or local recalibration may be necessary before deployment [54]. Similar issues may arise when models are transferred across countries, ECG machines, lead configurations, clinical settings, or populations with different disease prevalence.

Bias may also arise from the way outcomes are defined. Many AI-ECG models are trained using labels derived from EHRs, echocardiography reports, laboratory results, or billing codes. These labels can be incomplete or unevenly distributed because patients who undergo echocardiography or laboratory testing are not necessarily representative of the general population. This creates potential selection bias, especially for screening applications. In addition, disease prevalence strongly influences positive predictive value, so a model that performs well in a high-risk specialty cohort may generate many false positives when applied to a low-risk population.

External validation should evaluate not only discrimination, but also calibration, subgroup performance, predictive values at the intended disease prevalence, and whether the original decision threshold remains appropriate. Local recalibration may be necessary when populations, ECG hardware, or clinical settings differ from the derivation cohort, followed by continued monitoring for model drift after deployment. Reporting frameworks such as TRIPOD+AII [55], CONSORT-AI [56], SPIRIT-AI [57], and DECIDE-AI [58] improve transparency, but adherence to reporting standards does not itself establish clinical usefulness.

### 6.3. Data Leakage and Dataset Partitioning

Data leakage is an important methodological concern in AI-ECG research because individual patients may contribute multiple ECG recordings over time. If ECGs from the same patient appear in both the training and validation or test sets, a model may learn patient-specific characteristics rather than disease-specific patterns, potentially inflating estimates of model performance. Several major studies reviewed here minimized this risk through patient-level partitioning. Galloway et al. randomly partitioned patients into development and validation datasets for hyperkalemia detection [6], while Lima et al. separated patients into development and hold-out cohorts for ECG-based age estimation [7]. Similarly, the original Attia et al. study of left ventricular dysfunction evaluated its model in an independent test set of patients [4].

However, dataset partitioning is not reported with the same level of clarity across all AI-ECG studies. Some reports describe splitting data by date, by recording, or simply by “dataset” without stating whether ECGs from an individual patient could appear in more than one subset. Temporal or signal-level partitioning should not be assumed to provide patient-level independence unless explicitly documented [11]. Future AI-ECG studies should clearly report the unit of dataset partitioning and ensure that all ECGs from an individual patient remain within a single development, validation, or test set. This is particularly important in longitudinal ECG datasets containing repeated recordings from the same individuals.

### 6.4. Regulatory and Ethical Considerations

AI-ECG tools intended to influence diagnosis, screening, triage, or clinical management may fall under regulatory frameworks for medical device software. In the United States, the Food and Drug Administration has addressed artificial intelligence and machine learning-enabled software as medical devices through guidance on good machine learning practice, transparency, lifecycle management, and predetermined change control plans [59]. These frameworks are important because AI systems may evolve over time, may require updates after deployment, and may behave differently across populations or clinical environments.

In Europe, AI-ECG tools may be affected by both medical device regulation and the European Union Artificial Intelligence Act. The EU AI Act establishes requirements for high-risk AI systems, including risk management, data governance, transparency, human oversight, accuracy, robustness, cybersecurity, and post-market monitoring [60]. These requirements are highly relevant to AI-ECG because inaccurate or poorly monitored models could influence referral decisions, diagnostic testing, or treatment pathways [61].

Beyond regulatory compliance, ethical implementation requires human oversight, clear intended-use definitions, transparent outputs, and explicit responsibility for reviewing alerts, ordering confirmatory testing, and communicating results. AI-ECG should augment rather than override clinical judgment, particularly when model outputs concern conditions requiring imaging, laboratory testing, or hemodynamic confirmation [44].

Privacy and data security are also central to AI-ECG implementation. AI-ECG models are often developed using large repositories of ECG waveforms linked to EHR data, imaging results, laboratory values, and outcomes. These datasets must be governed by appropriate protections for patient privacy, secure data storage, controlled access, and compliance with relevant regulations. Cybersecurity is also important because compromised AI systems or data pipelines could affect clinical decision support, alter model outputs, or expose sensitive patient information.

Regulatory authorization does not eliminate institutional responsibility. Health systems must define the model’s intended use, the clinician responsible for acting on results, governance for software updates, safeguards for privacy and cybersecurity, and procedures for monitoring errors and unequal performance after deployment.

The principal challenges affecting the clinical implementation of AI-ECG and potential approaches to addressing them are summarized in Table 4.

## 7. Future Directions

### 7.1. Multimodal AI

One clear next step is multimodal modeling: combining the ECG waveform with other sources of clinical data. Single-modality AI-ECG has shown that the ECG carries information about cardiac structure, function, metabolism, and prognosis, but few real clinical decisions rest on the ECG alone. In practice, physicians integrate ECG findings with symptoms, physical examination, echocardiography, laboratory results, imaging, medications, and longitudinal history. Multimodal AI may better reflect this real-world decision-making process by combining complementary data streams into a single predictive framework.

ECG-based models such as EchoNext may support an ECG-to-imaging triage pathway by identifying patients most likely to have structural abnormalities on echocardiography [42]. True multimodal approaches may instead incorporate both ECG and echocardiographic information, as demonstrated in cardiac amyloidosis [33]. Both approaches must demonstrate that the added information improves clinical decision-making beyond ECG-only screening.

Multimodal AI may also strengthen risk prediction by incorporating EHR data. Variables such as age, sex, symptoms, comorbidities, medications, laboratory values, prior imaging, and previous ECGs may help refine AI-ECG predictions and improve calibration in specific clinical contexts. For example, a positive AI-ECG screen for ventricular dysfunction may have different implications in a young asymptomatic patient than in an older patient with dyspnea, diabetes, hypertension, and elevated natriuretic peptides. Combining ECG waveforms with EHR data could therefore improve risk stratification, reduce false positives, and help guide the most appropriate next diagnostic step.

The point at which ECG and EHR data are combined within the network is itself an important architectural consideration. In early, or input-level, fusion, clinical variables are combined with ECG inputs before substantial feature extraction, allowing interactions between modalities to be learned from the beginning but requiring harmonization of data with markedly different dimensionality and temporal resolution. In intermediate, or feature-level, fusion, separate encoders first transform ECG waveforms and EHR variables into learned representations that are subsequently concatenated or combined through mechanisms such as attention before final prediction. For example, Qiu et al. used separate ECG and clinical-data branches and combined their learned representations before final classification [62]. In contrast, late, or decision-level, fusion maintains separate modality-specific models and combines their predictions or risk assessments near the final decision stage. A related sequential deployment strategy is exemplified by TARGET-AI, in which longitudinal EHR information was used to identify higher-risk patients before selectively applying an AI-ECG model, reducing false-positive screening burden compared with untargeted screening [63]. Intermediate fusion may allow richer cross-modal interactions, whereas late fusion preserves modality-specific pathways and may make the contribution of each data source easier to audit. Importantly, current evidence does not establish one fusion strategy as universally superior; performance and clinical utility likely depend on the prediction task, temporal alignment and completeness of the data, computational requirements, and intended clinical workflow.

Another future application is longitudinal multimodal modeling. Instead of interpreting a single ECG in isolation, AI systems may eventually analyze serial ECGs together with trends in symptoms, laboratory results, imaging, medications, and clinical events. This could allow models to detect disease progression, treatment response, or early decompensation before conventional thresholds are reached. Such an approach may be particularly useful in heart failure, cardiomyopathy, pulmonary hypertension, chronic kidney disease, and other conditions in which risk evolves over time.

Multimodal models should not be assumed to outperform ECG-only systems simply because they use more data. Missing or asynchronous inputs, local EHR practices, and greater model complexity may reduce transportability and interpretability. Future studies should directly compare multimodal and ECG-only approaches and demonstrate incremental calibration, clinical decision benefit, workflow value, and patient outcomes. Until then, claims regarding treatment selection or response monitoring remain speculative.

### 7.2. Portable and Limited-Lead Technologies

Portable and limited-lead technologies may extend non-arrhythmic AI-ECG applications beyond conventional 12-lead acquisition. The reduced-lead hyperkalemia model discussed earlier demonstrated that clinically relevant metabolic information can be retained using only two or four ECG leads [6]. Separately, a prospective multicenter study showed that a deep-learning model applied to single-lead ECGs obtained during examination with an ECG-enabled stethoscope could identify patients with a left ventricular ejection fraction of 40% or lower [64]. These approaches could eventually expand AI-assisted screening in primary care, dialysis units, remote clinics, and settings with limited access to conventional diagnostic testing.

Portable deployment requires device-specific development and validation rather than direct transfer of a model trained on standard 12-lead ECGs. Reduced spatial information, variable device placement, motion artifact, and hardware differences may alter performance, while frequent alerts may increase false-positive testing. Studies should therefore evaluate each device in its intended setting and report diagnostic yield, confirmatory testing, access, diagnostic timing, and patient outcomes before portable AI-ECG is adopted for repeated monitoring.

Hardware constraints are another important consideration for wearable AI-ECG. Wearable and portable devices have limited memory, processing capacity, and battery power, making deployment of large deep-learning models challenging. Model-compression techniques such as quantization, pruning, and knowledge distillation can reduce model size and computational requirements, potentially enabling faster and more energy-efficient inference on low-power edge devices [65]. For example, Lee et al. compressed an ECG arrhythmia-classification model from 743 MB to 76 KB, an approximately 10,000-fold reduction, for embedded wearable deployment, reporting accuracies of 97.2% to 98.2% and inference times of approximately 150 to 600 ms across the model variants evaluated [66]. Although these results are promising, compression may introduce trade-offs between model size, inference speed, energy consumption, and diagnostic performance that should be evaluated for each intended device and clinical application.

### 7.3. Federated Learning

Federated learning may provide another pathway for expanding AI-ECG development across multiple institutions while limiting the need to centralize sensitive patient data. In a federated architecture, participating hospitals retain ECG and clinical data locally and train copies of a shared model on their own datasets; only model updates, rather than raw patient data, are transmitted to a coordinating server and aggregated into a global model [67]. This approach could allow AI-ECG models to learn from larger and more diverse populations while reducing barriers associated with sharing identifiable health data. ECG-specific studies have demonstrated the feasibility of federated training across heterogeneous datasets, including deep neural networks applied to raw 12-lead ECG signals [68]. However, federated learning does not eliminate all challenges: differences in patient populations, ECG acquisition systems, and disease prevalence across hospitals can complicate model convergence, while communication requirements and potential information leakage from model updates remain important considerations.

### 7.4. From Diagnostic Tool to a Digital Biomarker

The most speculative shift is from treating the ECG as a diagnostic test toward treating it as a digital biomarker. Historically, the ECG has been used to identify electrical abnormalities such as arrhythmias, conduction disease, ischemic changes, and repolarization abnormalities. AI-ECG analysis expands this role by generating quantitative outputs that may reflect broader physiologic states, including ventricular dysfunction, structural heart disease, metabolic abnormalities, biologic aging, and future mortality risk. In this context, the ECG is no longer only a snapshot of cardiac electrical activity; it becomes a data-rich physiologic signal that can be repeatedly measured, quantified, and linked to clinical outcomes.

As discussed above, ECG-age [7], mortality-prediction [8], and disease-specific models illustrate how AI-derived outputs may function as quantitative markers of physiologic risk rather than simple diagnostic labels. Their greatest future value may lie in repeated measurement, allowing changes in risk estimates to be tracked over time.

However, not every AI-ECG output should automatically be considered a clinically useful biomarker. A digital biomarker must have a reliable relationship to a biologic process, disease state, clinical outcome, or treatment response. More broadly, digital-biomarker frameworks emphasize analytical validity, clinical validity, reproducibility, and usefulness within a clearly defined clinical context [69]. In regulatory settings, the FDA similarly qualifies biomarkers for a specific context of use, particularly within medical-product and drug-development programs [70]. For AI-ECG, this means that model outputs should be reproducible across populations and ECG systems, externally validated, prospectively tested, and linked to clinical actions that improve outcomes or decision-making. Clinical use will require standardized reporting, defined thresholds, longitudinal validation, and safeguards against bias, model drift, and use outside the validated context.

AI-ECG outputs may ultimately function as digital biomarkers, but prognostic association alone is insufficient. Their value will depend on reproducible longitudinal measurement, a defined context of use, and evidence that changes in the output meaningfully guide clinical decisions or improve outcomes.

## 8. Conclusions

Deep learning has expanded the potential of the 12-lead ECG beyond conventional rhythm and waveform interpretation. The most mature evidence supports AI-ECG screening for reduced left ventricular ejection fraction, while applications involving structural heart disease, pulmonary hypertension, metabolic abnormalities, systemic disease, and broad prognostic assessment remain at different stages of validation.

Because ECGs are inexpensive and widely available, AI analysis may support earlier detection, triage, and selection for confirmatory testing. However, high discrimination does not by itself establish clinical benefit. Deployment requires external and prospective validation, appropriate thresholds for the intended population, defined follow-up pathways, monitoring for bias and model drift, and evidence that additional diagnoses lead to beneficial treatment or outcomes.

In the near term, AI-ECG is best viewed as clinical decision support rather than a replacement for imaging, laboratory testing, hemodynamic assessment, or physician judgment. Its broader role as a digital biomarker will depend on whether accurate predictions can be converted into reproducible and clinically actionable information.

## Figures and Tables

**Table 1:** Representative deep-learning architectures used in AI-ECG studies.

Principal Limitations	Principal Advantages	Representative Study/Application	Architecture
Long-range relationships are less explicit; limited interpretability	Learns local morphology and temporal features directly from raw multilead ECGs	Attia et al.—left ventricular dysfunction [4]	Convolutional neural network (CNN)
Limited interpretability	Captures temporal and cross-lead structural patterns	Ko et al.—hypertrophic cardiomyopathy [10]	Convolutional neural network (CNN)
Greater computational complexity	Multiple layers learn complex features; can use limited leads	Galloway et al.—hyperkalemia [6]	Deep CNN
Greater architectural complexity	Skip connections facilitate stable training of deeper networks	Lima et al.—ECG biological age [7]	Residual CNN (ResNet)
Computational burden and limited interpretability	Feature reuse and cross-lead attention weighting	Lin et al. (ECG12Net)—dyskalemia [11]	DenseNet-based network with attention

**Table 2:** Representative diagnostic performance reported for major AI-ECG applications.

Specificity (%)	Sensitivity (%)	AUC	Representative Study	Application
85.7	86.3	0.93	Attia et al. [4]	Reduced left ventricular ejection fraction
90	87	0.96	Ko et al. [10]	Hypertrophic cardiomyopathy
82.8	87.7	0.922	Siontis et al. [27] (overall analysis)	Hypertrophic cardiomyopathy (external validation)
74	78	0.85	Cohen-Shelly et al. [30] (ECG alone, test group)	Moderate/severe aortic stenosis
—	—	0.859–0.902	Kwon et al. [35]	Pulmonary hypertension
≈80	≈81	≈0.88	Liu et al. [36]	Elevated pulmonary artery pressure
—	—	0.853–0.883	Galloway et al. [6]	Hyperkalemia
—	—	>0.95 (hyperkalemia); >0.85 (hypokalemia)	Lin et al. [15]	Dyskalemia
—	—	0.88	Raghunath et al. [8]	One-year mortality
—	—	0.926 (internal); 0.883 (external)	Choi et al. [38]	Overt hyperthyroidism
65.9 (internal); 71.0 (external)	84.8 (internal); 77.4 (external)	0.854 (internal); 0.824 (external)	Yang et al. [40]	Anemia
—	—	0.901 (internal); 0.863 (external)	Kwon et al. [41]	Sepsis

Note: AUC, area under the receiver operating characteristic curve. Definitions of target conditions, diagnostic thresholds, cohort composition, and validation design differed among studies; performance estimates should therefore not be compared directly across rows. Values marked ‘≈’ are approximate, and em dashes indicate that the metric was not reproduced here; readers should consult the primary publication for complete performance reporting.

**Table 3:** Major clinical applications of AI-enabled electrocardiography.

Application	Representative Findings	Potential Clinical Role	Representative Studies
Reduced left ventricular ejection fraction	Large studies demonstrated strong discrimination, and the EAGLE trial showed increased detection and targeted echocardiography.	Screening and selection for confirmatory echocardiography.	Attia et al. [4]; Yao et al. (EAGLE) [19]
Structural heart disease	Models have detected hypertrophic cardiomyopathy, valvular disease, and cardiac amyloidosis, with external validation reported for several applications.	Identification of patients who may benefit from confirmatory imaging or disease-specific testing.	Ko et al. [10]; Cohen-Shelly et al. [30]; Goto et al. [33]
Pulmonary hypertension	Multiple models demonstrated good discrimination and potential detection before formal diagnosis.	Earlier echocardiography, specialist referral, and hemodynamic evaluation.	Kwon et al. [35]; Liu et al. [36]; DuBrock et al. [37]
Dyskalemia	Hyperkalemia and hypokalemia models have shown strong performance in large cohorts using laboratory potassium as the reference standard.	Rapid screening and prioritization of confirmatory laboratory testing.	Galloway et al. [6]; Lin et al. [15]
Other systemic conditions	Exploratory models have been developed for hyperthyroidism, anemia, sepsis, and dyscalcemia.	Identification of patients requiring targeted laboratory or clinical evaluation.	Lin et al. [16]; Choi et al. [38]; Yang et al. [40]; Kwon et al. [41]
Biologic age and mortality risk	AI-derived ECG age and mortality models identify prognostic information that may not be visually apparent.	Risk stratification and possible longitudinal monitoring.	Lima et al. [7]; Raghunath et al. [8]

**Table 4:** Major challenges in clinical implementation of AI-enabled electrocardiography.

Challenge	Clinical Significance	Potential Approach
Limited interpretability	Clinicians may be uncertain whether predictions reflect meaningful physiology or artifacts.	Use explainability tools cautiously alongside external validation, calibration, and failure-mode testing.
Dataset bias and limited generalizability	Performance may differ across populations, clinical settings, ECG systems, and disease prevalence.	Perform external and subgroup validation, local recalibration, and continued monitoring for model drift.
False-positive and false-negative results	Incorrect classifications may lead to unnecessary testing or inappropriate reassurance.	Select thresholds for the intended population and connect results to defined confirmatory pathways.
Workflow integration and alert fatigue	Accurate predictions may be ignored when responsibility and follow-up are unclear.	Define how alerts are delivered, who reviews them, and what clinical action should follow.
Regulatory, ethical and privacy concerns	AI systems may affect clinical decisions while using large, linked health datasets.	Establish human oversight, data-security safeguards, update governance, and post-deployment monitoring.

## Data Availability

No new data were created or analyzed in this study. Data sharing is not applicable to this article.
